# Human Adipose-Derived Stem/Stromal Cells Promote Proliferation and Migration in Head and Neck Cancer Cells

**DOI:** 10.3390/cancers13112751

**Published:** 2021-06-01

**Authors:** Kariem Sharaf, Tanja K. Eggersmann, Stefan P. Haider, Sabina Schwenk-Zieger, Jiefu Zhou, Olivier Gires, Axel Lechner, Martin Canis, Frank Haubner

**Affiliations:** 1 Department of Otolaryngology, University Hospital, LMU Munich, Marchioninistrasse 15, 81377 Munich, Germany; Stefan.Haider@med.uni-muenchen.de (S.P.H.); Sabina.Schwenk-Zieger@med.uni-muenchen.de (S.S.-Z.); Jiefu.Zhou@med.uni-muenchen.de (J.Z.); Olivier.Gires@med.uni-muenchen.de (O.G.); Axel.Lechner@med.uni-muenchen.de (A.L.); Martin.Canis@med.uni-muenchen.de (M.C.); 2 Department of Obstetrics and Gynecology, University Hospital, LMU Munich, Marchioninistrasse 15, 81377 Munich, Germany; Tanja.Eggersmann@med.uni-muenchen.de; 3 Department of Gynecological Endocrinology and Reproductive Medicine, University Hospital of Schleswig-Holstein, 23562 Lübeck, Germany; 4 Clinical Cooperation Group “Personalized Radiotherapy in Head and Neck Cancer“, Helmholtz Zentrum München, 85764 Neuherberg, Germany

**Keywords:** MSC, ASCs, head and neck squamous cell carcinoma, esophageal squamous cell carcinoma, regenerative medicine

## Abstract

**Simple Summary:**

Fat grafts obtained from a minimal invasive liposuction device contain multipotent stem cells termed adipose-derived stem/stromal cells (ASCs). ASCs can be used for their proposed wound healing relevant characteristics, including for tissue defects in cancer patients. For head and neck cancers, little is known about the effects of ASCs on tumor cells. Using supernatants of ASCs from five patients in different functional experiments, this study aimed to investigate how ASCs influence tumor growth, invasive properties, and neoangiogenesis. The data show that all mentioned characteristics are promoted by fat graft stem cells in vitro in head and neck cancer cell lines. Although clinical relevance of these in vitro findings is unclear, due to the lack of in vivo and clinical data, fat grafts should be used cautiously and complete removal of tumor should be ensured before augmentation in head and neck cancer patients is performed.

**Abstract:**

Human adipose-derived stem/stromal cells (ASCs) are increasingly used as auto-transplants in regenerative medicine to restore tissue defects or induce wound healing, especially in cancer patients. The impact of ASCs on squamous cell carcinoma of the upper aerodigestive tract (UAT) including head and neck and esophageal squamous cell carcinoma (HNSCC and ESCC) is not yet fully understood. ASCs were cultured from subcutaneous, abdominal lipoaspirates of five patients, who received auto-transplants to the head and neck. Supernatants were tested for paracrine effects in functional in vitro assays of proliferation of HNSCC tumor cell line FaDu and ESCC cell line Kyse30, and their cell migration/invasion capacities in Boyden chambers, in addition to endothelial tube formation assay using human umbilical vein endothelial cells (HUVECs). All ASC-derived supernatants enhanced proliferation of FaDu cells, invasive migration, and tube formation by HUVECs, compared to controls. Of five patients’ lipoaspirates, ASC-derived supernatants of four patients increased proliferation and invasive migration in Kyse30 cells. The data suggests that ASCs can promote tumor cell proliferation, invasiveness, and neo-angiogenesis in these tumor cell lines of the UAT and HUVEC in a paracrine manner. Although clinical studies on the subject of oncological safety are still needed, these findings emphasize the importance of complete tumor removal before ASCs are used in the head and neck.

## 1. Introduction

Stem cell-based therapies offer a new approach for the repair of volume defects and support wound healing. The work with either embryonic stem cells or induced pluripotent stem cells is highly restricted in the clinical setting in most countries due to regulations and ethical discourse, even though these cells are theoretically highly advantageous [1,2]. Mesenchymal stromal cells (MSCs) are non-hematopoietic, pluripotent progenitor cells that have been shown to reside in various tissues and organs, such as adipose tissue, umbilical cord matrix, synovial membrane, and dental tissues [3,4]. Because the isolation of MSCs from adipose tissue is comparably uncomplicated, adipose-derived stem cells (ASCs, also adipose-derived stromal cells) are one of the most widely used types of stem cells in clinical application to date [5]. Adipose tissue has been used for tissue augmentation since 1893, first performed by the German surgeon Neuber to fill a depressed scar on the face [6].

ASCs are localized in the perivascular niche of adipose tissues and are considered a suitable source for stem cell-based therapies, mainly because they can be collected in significant quantities during surgical fat transfer or minimally invasive procedures with techniques such as lipoaspiration [7,8,9]. In contrast to, e.g., breast augmentation in plastic surgery, which uses large amounts of lipoaspirates, only small volumes are usually required in head and neck surgery [9,10]. The volumes of lipoaspirates that are needed for procedures such as vocal fold augmentation typically amount to a few milliliters [11]. ASCs are part of the stromal vascular fraction that is extracted after enzymatic digestion (or mechanical dissociation) and centrifugation of adipose tissue [12,13].

The wound healing properties of ASCs are mostly attributed to their paracrine effects [13,14]. Soluble growth factors and cytokines secreted by ASCs, such as Vascular Endothelial Growth Factor (VEGF) and Transforming Growth Factor Beta 3 (TGF-β3), modulate various aspects of the wound healing process such as neovascularization, homing of stromal cells, and the inhibition of fibrosis [13,15]. Some experimental studies suggest that ASCs are responsible for tumorigenesis and tumor development promoted by inflammatory cytokines that are secreted from the ASCs [16,17,18]. Further studies hypothesized that adipocyte and ASC secretions can stimulate tumor cell growth and that tumor–stroma interaction can potentially trigger cancer recurrence by activating residual cancer cells in the tumor bed [19,20].

Generally, adipose tissue transplantation is considered a safe procedure in different clinical settings, especially in cancer patients [21,22,23]. Head and neck squamous cell carcinoma (HNSCC) patients have frequent wound healing difficulties in many stages of their diseases [11,24]. Although ASCs facilitate wound healing, they may accelerate recurrence of cancer when applied to a localization with residual tumor cells [22]. Women with a history of breast cancer often desire reshaping or breast augmentation after surgery and autologous fat grafting is an established and frequently performed therapy in these patients [25]. Therefore, many studies have focused on oncological safety of autologous fat grafting in breast cancer patients and few studies have tried to examine effects and safety in other tumor entities [26,27]. To date, the data on the interaction of ASCs and breast cancer cells, and the oncological safety of ASCs in breast cancer patients, remain inconclusive [18,25,27,28,29,30,31]. Although many experimental studies showed that ASCs were able to counteract cancer therapy or promote tumorigenesis in breast cancer cells, meta-analysis of clinical studies found no differences in recurrence rates of breast cancer in patients treated with grafts or not [25,29,30]. For bladder cancer, in vitro data has shown that ASCs might promote proliferation and invasive properties of cancer cells [32].

To date, only eight studies have linked MSCs/ASCs and HNSCC [24,33,34,35,36,37,38,39]. Grønhøj et al. evaluated the salivary gland function after ASC injection in patients with radiation-induced xerostomia after HPV-related oropharyngeal squamous cell carcinoma, but oncological safety was not an endpoint [24]. Xia et al. concluded that gingiva-derived MSCs inhibit tumorigenesis of oral cavity squamous cell carcinoma of the tongue [33]. Chiu et al. tested paracrine effects of ASC-conditioned medium on oral squamous cell carcinoma and found that it might protect cancer cells from cisplatin-induced cell death but did not find alterations in tumor cell growth and migration [34]. In different in vitro approaches and an in vivo xenograft model, Danan et al. found that ASCs did not significantly stimulate HNSCC cell proliferation or migration, and only increased survival in one single cancer cell line tested [35]. Rowan et al. did not find alterations in growth of HNSCC cells in vitro or tumor xenografts ex vivo, but found stimulation of cancer cell migration, integration of ASCs to tumor–stroma, and early micrometastasis in vivo [36]. Using bone marrow-derived MSCs of different donors and different HNSCC cell lines, the data of Wessely et al. suggested increased invasion properties and prometastatic effects of MSCs on HNSCC [37]. In the most recent study and in agreement with an older study of their group, Sinha et al. found that bone marrow-derived MSCs but not ASCs contribute to tongue squamous cell carcinoma progression [38,39].

In summary, these studies show an inhomogeneous state of knowledge regarding the role of ASCs in HNSCC progression. It remains unclear how ASCs and HNSCC cells interact and how underlying mechanisms might facilitate tumor progression in HNSCC. In this project, we aimed to characterize the paracrine effects of ASCs on representative cancer cell lines of the UAT. Therefore, we performed experiments with supernatants from ASCs that were acquired from lipoaspirate samples of five patients and tested their influence on HNSCC cell line FaDu and ESCC cell line Kyse30. To gain further insights in the interaction of ASC and UAT cancers, different assays were performed to assess tumor cell proliferation, invasive properties, and the ability to grow new blood vessels under the influence of ASCs in vitro.

## 2. Materials and Methods

### 2.1. Patient Characteristics

Patients included in this study underwent elective autologous fat grafting to the head and neck area (*n* = 5). General patient characteristics of these five patients are summarized in Table S1.

All samples were obtained after written informed consent during routine surgery. The study was conducted according to the guidelines of the Declaration of Helsinki and approved by the institutional ethics committee of the local medical faculty (Ethikkommission der Medizinischen Fakultät der Ludwig-Maximilians-Universität, IRB approval number 17-567).

### 2.2. Acquistion of Lipoaspirates

Lipoaspirates were obtained from the abdominal fat tissue (*n* = 5) with a thin (2 mm diameter) liposuction cannula (Spiggle&Theis, Overath, Germany) as described previously [9]. Lipoaspirates were then centrifugated for 5 min at 3000 rpm and the resulting oily and saline fractions were discarded, and the stromal vascular fraction was transferred to 1 mL syringes for injection into the augmentation site [9]. A small amount was transferred to the laboratory facilities.

### 2.3. Explant Cultivation and Isolation of Adipose-Derived Mesenchymal Stem Cells

As described previously [40], lipoaspirate tissue was washed three times with 37 °C pre-warmed PBS (Apotheke Klinikum der Universität München, Munich, Germany) and centrifuged for 7 min at 900 rpm (Varifuge 3.OR, Heraeus, Hanau, Germany, rotor diameter = 21.5 cm). The resulting liquid phase and oily supernatants were discarded. The remaining cellular fraction was plated on 100 mm tissue culture dishes (Cell+, Sarstedt, Nürmbrecht, Germany). Approximately 1 mL of lipoaspirate was plated per dish. For better attachment, the tissue was left for 5 min before carefully adding 2.5 mL of pre-warmed Mesenchymal Stem Cell Growth Medium 2 (MSCM 2, PromoCell, Heidelberg, Germany) to each tissue culture plate. Thereafter, plates were maintained at 37 °C in a humidified atmosphere with 5% CO_2_. On the third day of cultivation, another 2 mL of MSCM 2 was added. On the sixth day, the conditioned medium was collected, centrifuged, and frozen. Hereafter, the medium was changed three times per week. Between day 12 and 14, the tissue was carefully removed and tissue culture plates were washed thoroughly with pre-warmed PBS. To test the MSC characteristics as described before, immunophenotyping of these cultured cells and differentiation assays were performed [13].

### 2.4. Acquistion of ASC-Derived Supernatants

At a partial confluency of 80–90%, cells were detached using a 0.5% trypsin/0.2% EDTA solution (Biochrom GmbH, Berlin, Germany), and further cultivated in tissue culture flasks (Sarstedt). After meeting the criteria for MSC as described before, these cells were referred to as passage 0 ASCs and were passaged [13]. In passage 1, the conditioned media were collected after 72 h of cell culture, centrifuged, and frozen.

### 2.5. HNSCC Tumor Cell Lines and Proliferation Assay

FaDu and Kyse30 cell lines were obtained from Leibniz Institute German Collection of Microorganisms and Cell Culture (DSMZ). Cells were maintained in DMEM (FaDu) or RPMI 1640 (Kyse30), 10% FCS, 1% penicillin/streptomycin, in a 5% CO_2_ atmosphere at 37 °C. In the proliferation assay, 10^5^ tumor cells were seeded in 6-well plates, washed with PBS after 24 h and incubated with ASC-derived supernatants from the five above mentioned individual patients as verum treatments or (1) supernatant from FaDu/Kyse30 with MSCM 2, (2) DMEM/RPMI without FCS, or (3) DMEM/RPMI with FCS as controls (1–3). After 72 h, cells were counted in an EVE automatic cell counter (Nano Entek, VWR, Munich, Germany). Supernatant from FaDu/Kyse30 with MSCM was defined as the baseline control for the ASC-derived supernatants, and therefore, all collected data were normalized to the mean of this control.

### 2.6. Cell Migration Assay

To investigate cell migration over a microporous membrane, a “Boyden chamber” assay with an 8 µm pore size (QCM 24-Well Colorimetric Cell Migration Assay, Merck, Darmstadt, Germany) was performed [41,42]. A quantity of 2.5–3.0 × 10^6^ FaDu or Kyse30 cells in 300 µL DMEM/RPMI without FCS were placed into the upper inserts, and ASC-derived supernatants from the five above mentioned individual patients as verum treatments or (1) supernatant from FaDu/Kyse30 with MSCM 2, (2) DMEM/RPMI without FCS, or (3) DMEM/RPMI with FCS as controls (1–3) were placed in the lower wells and incubated for 24 h. After incubation for 24 h, fluid of the lower well was analyzed using a colorimeter (VersaMax Microplate Reader, Molecular Devices, San José, CA, USA).

The DMEM/RPMI without FCS control was used as a “blank control” and its resultant values were subtracted from the corresponding verum or control experiment results automatically. The supernatant from FaDu/Kyse30 with MSCM 2 control was defined as the baseline control for the ASC-derived supernatants, and, therefore, all data were normalized to the mean of this control.

### 2.7. Neo-Angionesis Assay

To mimic angiogenesis, a tube formation assay was performed as described previously [43,44]. Matrigel Matrix GFR (Corning, Fisher Scientific, Schwerte, Germany) was added to a µ-plate angiogenesis 96-well (ibidi, Gräfelfing, Germany) following the manufacturer’s application guide. Human umbilical vein endothelial cells (HUVECs, PromoCell, Heidelberg, Germany) in passage 3–5 were grown to 70–80% confluence, trypsinated with trypsin-EDTA (Thermo Scientific, Schwerte, Germany), and centrifuged at 300× *g* for 5  min. Subsequently, cells were resuspended in either the ASC-derived supernatants (cultured with MSCM 2), fresh Endothelial Cell Growth Medium MV (ECGM, PromoCell, Heidelberg, Germany), fresh MSCM 2 (PromoCell, Heidelberg, Germany), or ECGM with suramin (30 nM, PromoCell, Heidelberg, Germany). Then, 15,000 cells/well were seeded into an ibidi μ-slide angiogenesis 96-well plate and incubated at 37 °C, 5% CO_2_, and 95% humidity. Photographs of each well were taken after 20 h with a Leica DMi8 microscope (Leica, Wetzlar, Germany) using 5× magnification and phase contrast Ph (PLAN 5X/0.12 DRY). Each group consisted of at least three technical replicates of each experiment; results from three independent experiments are shown. ImageJ “Angiogenesis Analyzer” was used to analyze the photographs of the tube formation assay. Here, branches were identified and calculated.

### 2.8. Statistical Analysis

R version 3.6.0 was utilized for statistical analysis [45]. For each assay type, we performed a Kruskal–Wallis H test to determine if differences between verum and control groups were present (“kruskal.test” function, R “stats” package [45]), followed by Wilcoxon rank sum test-based pairwise comparisons with Benjamini Hochberg p-value adjustment (“wilcox.test” and “p.adjust” functions, R “stats” package [45]). *p*-values < 0.05 indicated statistical significance.

## 3. Results

### 3.1. ASC-Derived Supernatants Promote UAT Tumor Cell Proliferation In Vitro

**Figure 1:**
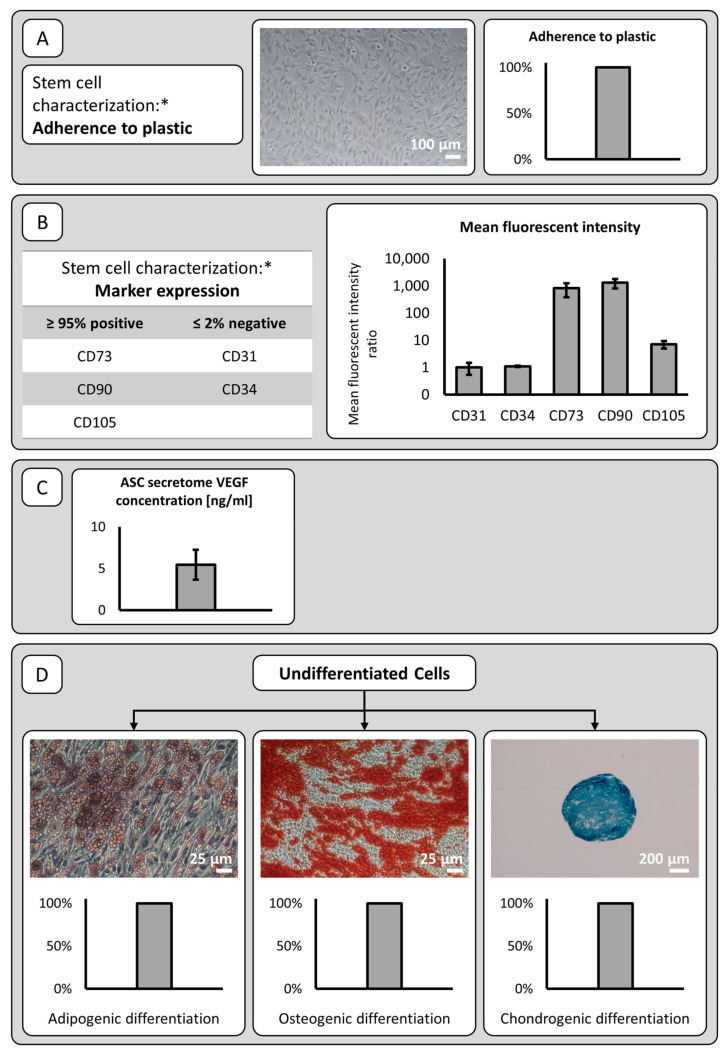
Mesenchymal stromal cell characterization was performed as described previously (*) [13]: (**A**) Mean Plastic adherence was found in all patient cell lines. A representative photograph is shown. (**B**) Cell lines were tested for positivity or negativity of the indicated markers using flow cytometry of passage 2 cells. All patient cell lines were positive for CD73, CD90, and CD105 and negative for markers CD31 and CD34 (mean fluorescent intensity (MFI) ± SD), as expected for mesenchymal stromal cells. (**C**) ELISA results for VEGF in ASC culture supernatants collected in passage 1 are shown. Results for cell culture supernatants are normalized to ng/106 cells (mean ± SD). (**D**) Lipid vesicle accumulation visualized by Oil Red O staining in adipogenic differentiated ASCs after two weeks of incubation with adipogenic differentiation medium (magnification 200×). Alizarin S staining of extracellular calcium deposits in osteogenic differentiated ASCs after two weeks of incubation with osteogenic differentiation medium (magnification 200×). Alcian blue staining of cartilage extracellular matrix in ASCs cultured as three-dimensional spheroids after four weeks of incubation with chondrogenic differentiation medium (magnification 40×). Shown are representative images of *n* = 5 independent experiments.

Initially, we characterized the cell lines obtained from lipoaspirates of five patients and assessed their stem cell capacities as described before [13]. Three of the five cell lines had already been characterized previously [13]. Cells from all five patients were characterized as ASCs. The scheme used to characterize cell lines is shown in Figure 1.

**Figure 2:**
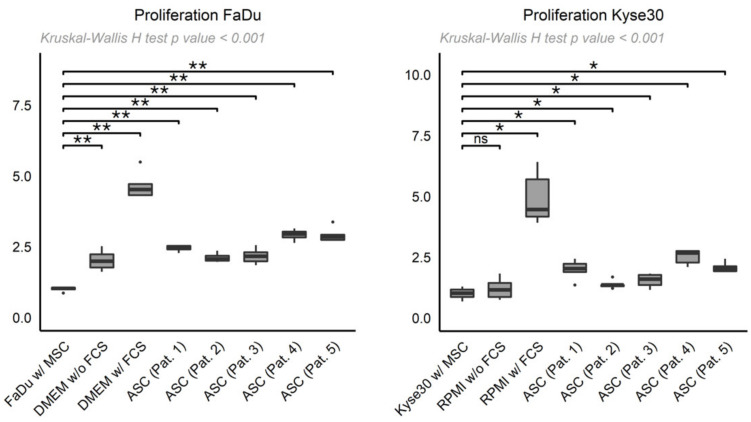
Box-whisker plots depicting normalized proliferation of FaDu (**left**) and Kyse30 cells (**right**), cultured with ASC-derived supernatants from patients 1–5 (“ASC (Pat. 1–5)”) or supernatant from FaDu/Kyse30 with MSCM 2 (“FaDu w/ MSC”, “Kyse30 w/ MSC”), DMEM/RPMI without FCS (“DMEM w/o FCS”, “RPMI w/o FCS”), and DMEM/RPMI with FCS (“DMEM w/ FCS”, “RPMI w/ FCS”) as controls. Shown are mean ± SD from *n* = 3 independent experiments. Statistical test: Kruskal–Wallis H test, *p*-values * < 0.05, ** < 0.01.

To assess the paracrine function of these ASCs on UAT tumor cell proliferation, FaDu and Kyse30 cells were cultured with ASC-derived supernatants of the five patients or controls. The mean ± SD FaDu cell proliferation rate was approximately increased in a range of 2.10- to 2.92-fold compared to a matched negative control (Figure 2; Patient 1: 2.44 ± 0.10, patient 2: 2.10 ± 0.15, patient 3: 2.16 ± 0.26, patient 4: 2.92 ± 0.19, patient 5: 2.90 ± 0.25 vs. FaDu supernatant: 1.00 ± 0.07; all *p* < 0.01, Wilcoxon rank sum test). In addition, the FaDu cells cultured with positive controls also demonstrated increased proliferation rates (DMEM without FCS: 2.01 ± 0.34, DMEM with FCS: 4.64 ± 0.46 vs. FaDu supernatant; both *p* < 0.01, Wilcoxon rank sum test).

In Kyse30 cells, the mean ± SD proliferation rate was increased in the presence of ASC-derived supernatants in a range of 1.37- to 2.52-fold compared to a negative control (Figure 2; Patient 1: 1.98 ± 0.41, patient 2: 1.37 ± 0.16, patient 3: 1.53 ± 0.31, patient 4: 2.52 ± 0.31, patient 5: 2.07 ± 0.20 vs. Kyse30 supernatant: 1.00 ± 0.23; all *p* < 0.05, Wilcoxon rank sum test). As already seen in FaDu cells, the highest proliferation rate in Kyse30 was found in a positive control (RPMI with FCS), whereas the RPMI control without FCS showed no significant difference (RPMI with FCS: 4.90 ± 1.06 vs. Kyse30 supernatant, *p* = 0.01; RPMI without FCS: 1.19 ± 0.42 vs. Kyse30 supernatant, *p* = 0.47, Wilcoxon rank sum test).

Overall, HNSCC tumor cell proliferation was significantly increased following incubation with ASC supernatants from five different patients compared to the respective negative control conditions.

### 3.2. ASC-Derived Supernatants Promote Invasion of HNSCC Tumor Cells in a Functional In Vitro Assay

To investigate cell migration of HNSCC over an 8 µm microporous membrane, we performed a colorimetric Boyden chamber assay according to the proliferation experiments above. After subtraction of the negative control results (DMEM without FCS), the data was again normalized and compared to the tumor cell supernatant control.

**Figure 3:**
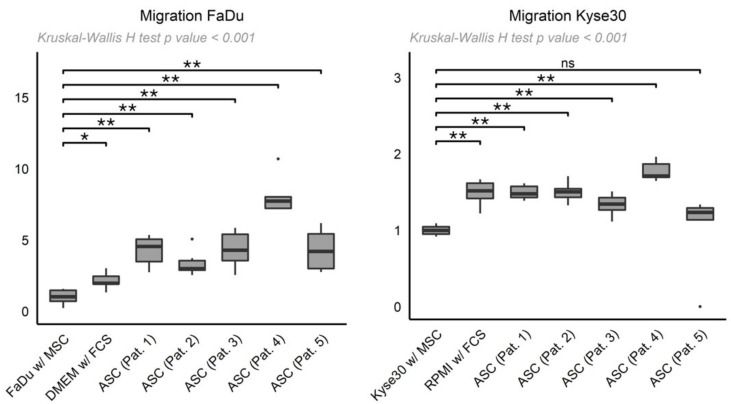
Box-whisker plots depicting migration (normalized after subtraction of negative controls) of FaDu (**left**) and Kyse30 cells (**right**) towards ASC-derived supernatants from patients 1–5 (“ASC (Pat. 1–5)”) or supernatant from FaDu/Kyse30 with MSCM (“FaDu w/ MSC”, “Kyse30 w/ MSC”), and DMEM/RPMI with FCS (“DMEM w/ FCS”, “RPMI w/ FCS”) as controls. Shown are mean ± SD from *n* = 3 independent experiments. Statistical test: Kruskal–Wallis H test, *p*-values * < 0.05, ** < 0.01, ns: not significant.

Here, FaDu cells migrated more strongly towards all five ASC-derived supernatants and the positive control (DMEM with FCS) than towards FaDu cell supernatants (Figure 3; Patient 1: 4.25 ± 1.08, patient 2: 3.36 ± 0.92, patient 3: 4.33 ± 1.29, patient 4: 8.16 ± 1.46, patient 5: 4.29 ± 1.48, DMEM with FCS: 2.12 ± 0.60 vs. FaDu supernatant: 1.00 ± 0.54; all *p* < 0.01 except DMEM with FCS: *p* = 0.01, Wilcoxon rank sum test).

In Kyse30 experiments, cancer cells showed higher migration towards four of the five ASC-derived supernatants and the positive control (RPMI with FCS) compared to migration towards Kyse30 supernatant (Figure 3; Patient 1: 1.49 ± 0.09, patient 2: 1.50 ± 0.13, patient 3: 1.33 ± 0.14, patient 4: 1.77 ± 0.13, patient 5: 1.04 ± 0.51, RPMI with FCS: 1.49 ± 0.17 vs. Kyse30 supernatant: 1.00 ± 0.07; all *p* < 0.01 except patient 5: *p* = 0.07, Wilcoxon rank sum test).

In conclusion, HNSCC tumor cells migrated more strongly towards ASC-derived supernatants than cognate controls. In concordance with the proliferation experiments, this effect was more pronounced in FaDu cells than Kyse30 cells.

### 3.3. ASC-Derived Supernatants Promote Neo-Angiogenesis in a Functional Endothelial Tube Formation Assay In Vitro

To mimic neo-angiogenesis, we assessed the influence of ASC-derived supernatants and respective controls on tube formation in a functional endothelial cell assay. HUVECs were incubated in the presence of ASC supernatants or the indicated controls and the number of branches during tube formation was assessed after 20 h. After subtraction of branch numbers of the negative control (ECGM with 30 nM Suramin), the resulting branch number data was normalized and compared to the MSCM2 control group mean.

**Figure 4:**
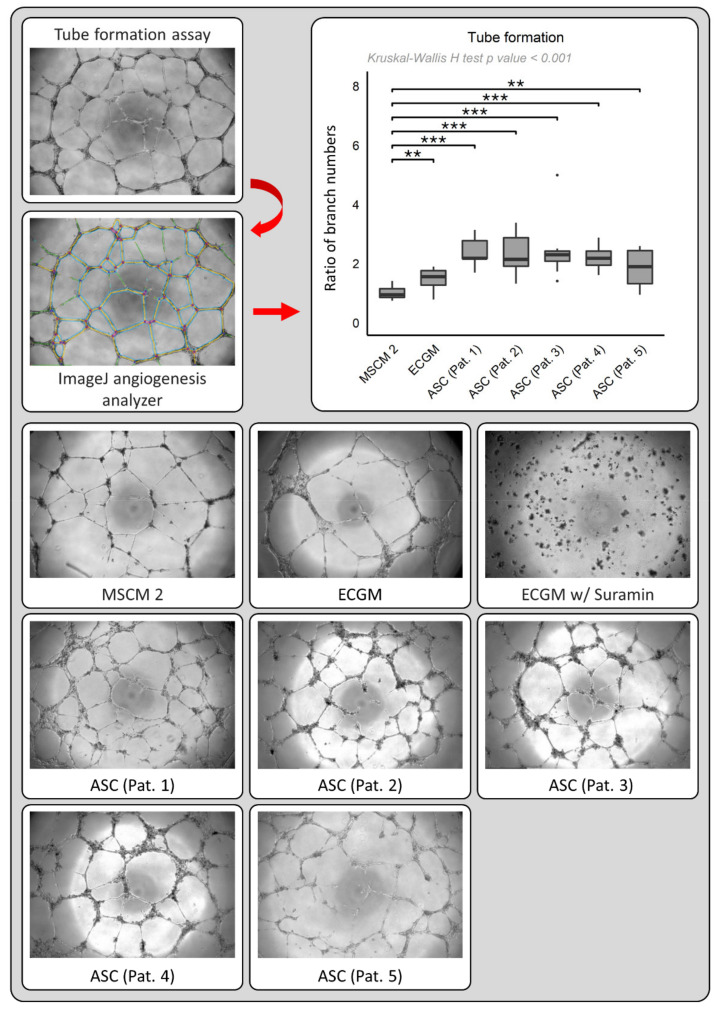
Top left: Branches (yellow lines) in each well were counted using ImageJ Angiogenesis Analyzer software. Top right: Box-whisker plots depicting tube formation (number of branches normalized to MSCM 2 control after subtraction of ECGM with 30 nM Suramin control) of HUVECs cultured with the MSCM 2 control, ECGM (without Suramin) control, or ASC-derived supernatants from five different patients (“ASC (Pat. 1–5)”). Lower half: Representative photographs depicting tube formation in wells corresponding to patients 1–5 (“ASC (Pat. 1–5)”) and controls. *p*-values ** < 0.01, *** < 0.001.

Representative photographs of tube formation experiments are depicted in Figure 4. In comparison to the control group (MSCM 2), the mean ± SD number of branches in the tube formation assay was increased 2- to 3-fold when cultured with ASC-derived supernatants of the five patients, and increased approximately 1.5-fold when cultured with ECGM (Figure 4; Patient 1: 2.37 ± 0.54, patient 2: 2.31 ± 0.68, patient 3: 2.45 ± 1.02, patient 4: 2.21 ± 0.43, patient 5: 1.83 ± 0.66, ECGM: 1.48 ± 0.36 vs. MSCM 2: 1.00 ± 0.21; Patient 1–4: *p* <0.001, patient 5 and ECGM: *p* < 0.01, Wilcoxon rank sum test).

In conclusion, HUVECs built more branches in a functional tube formation assay under the influence of ASC-derived supernatants than various cell mediums including growth-promoting supplements.

## 4. Discussion

Generally, adipose tissue transplantation is used successfully in various indications, e.g., wound healing, cardiovascular, and even joint disorders [46]. In the head and neck area, adipose tissue transplantation belongs to the most common topical stem cell therapies [11,47]. For augmentation of volume defects and treatment of impaired wound healing, adipose tissue transplantation is an established regenerative and reconstructive approach in head and neck cancer patients [48]. Therefore, ASCs are injected into tissues that might still contain residual tumor cells; in the head and neck area, these are most likely from squamous cell carcinoma [35,49]. In a literature search using the search terms “ASC AND HNSCC”, “adipose stem cells AND HNSCC”, “MSC AND HNSCC” and “mesenchymal stem cells AND HNSCC”, we found only six studies that examined the role of ASCs on HNSCC progression [33,34,35,36,37,38]. Their results are controversial and support both the notion that ASCs do support HNSCC progression and the opposing assumption that ASCs do not alter HNSCC progression. Our literature search of ASC/MSC and ESCC (search terms esophageal cancer AND ASC/adipose stem cells/MSC/mesenchymal stem cells) did not find a study that directly investigated ASC/ESCC interactions. The closest example was a study that showed tumor-promoting properties of adipose tissue on ESCC [50].

The present study focused on the effects of ASCs on two different cancer cell lines: FaDu cells represent a cell line gained from HNSCC of the hypopharynx with an estimated doubling time of 50 h; Kyse30 cells are derived from an esophageal well differentiated squamous cell carcinoma (doubling time 30 h). Both are well-characterized squamous cell carcinoma cell lines and represent established cancer cell lines of the UAT [51,52].

According to our data, ASC-derived supernatants promote tumor cell proliferation of FaDu and Kyse30 cells using conditioned media from ASC. Overall, tumor cells grew significantly faster than under respective negative control conditions in all combinations of two different cancer cell lines and ASC-derived supernatants obtained from five different patients. Although our results on tumor cell proliferation are unambiguous, in contrast, none of the other studies found increased HNSCC proliferation when ASC-conditioned medium or ASCs were added [34,35,36,38], except for increased colony formation only in SCC9 cells [35]. Although not testing directly, Nakayama et al. hypothesized that adipose tissue might increase ESCC tumor growth and decrease apoptosis of ESCC [50]. Although it remains unclear why most experiments did not show increased tumor cell growth in HNSCC, our experiments with ESCC cell line Kyse30 support the hypothesis of Nakayama.

The promotion of cell growth and invasiveness are typical attributes of aggressive tumor behavior. This behavior was shown in a study by Su et al., who examined tumor-promoting effects of ASCs on prostate cancer cells [53]. To evaluate the invasion capacity of HNSCC tumor cells stimulated with ASC supernatants, a migration assay using microporous membranes was used. This assay revealed that ASC-derived supernatants promote migration of HNSCC tumor cells in vitro towards the ASC-derived supernatants. This finding is of outmost importance with respect to dormant residual tumor cells, which might be re-activated by the ASC secretome. According to proliferation experiments of our study, this effect was more pronounced in FaDu cells than Kyse30 cells. This might result from the different doubling times of the cell lines with the doubling time of Kyse30 cells already being short and, therefore, less prone to the impact of the ASC secretome [51,52].

In the literature about HNSCC–ASC interactions, pro-migratory effects of the ASC secretome were found in the study of Rowan et al. [36], whereas three other studies did not find effects on migration or invasiveness [34,35,38]. However, there are findings that indicate carcinogenic effects of ASCs in those afore-mentioned studies: ASC-conditioned medium triggered chemoresistance of SCC25 and Cal27 cells, possibly through upregulation of the IGF-1R/AKT/ERK signaling pathway [34]. Also using Cal27 cells, Rowan et al. showed that ASCs were built into the tumor–stroma when Cal27 cells and ASCs were co-injected into the flank of nude mice and promoted HNSCC micrometastases to the murine brain [36]. Moreover, our results on invasiveness are similar to results of groups that had studied interactions of bone marrow-derived MSCs and HNSCC [37,39], and bone marrow-derived MSCs and ESCC [54,55,56].

In general, various studies have discussed the safety of ASC applications in cancer patients because the pursued regenerative properties of MSCs might promote tumor relapse [22]. Therefore, another important finding of our study is that ASC-derived supernatants promote neo-angiogenesis in a functional endothelial cell tube formation assay, in addition to their tumorigenic effects on HNSCC. Angiogenesis and neovascularization are highly complex processes, regulated by multiple players. Vascular endothelial growth factor (VEGF) is a central component in this context because it regulates neovascularization by targeting endothelial cell migration and proliferation [57]. In a previous study of our group, we found elevated VEGF levels in the secretome of ASCs and were able to confirm this finding in the five ASC cultures used in this work [13]. VEGF and other soluble factors of the secretome likely explain the pro-angiogenetic effects of ASCs. In addition to the paracrine effects of ASCs on angiogenesis, the literature also describes direct differentiation of ASCs into endothelial cells [58]. The exact mechanisms depend most likely on the maturation and stage of differentiation of ASCs [59]. Regarding neo-vascularization in a potentially dormant tumor site independent from stem cell therapies, local and/or free flap surgery is a commonly accepted and well-studied reconstructive method, in which a well-vascularized tissue flap is in direct contact with the tumor bed and extensive local vascular remodeling occurs. Immediate autologous tissue flap reconstruction has not been correlated with increased recurrence rates, e.g., in breast cancer patients [60,61]. Therefore, increased local vascularization at the site of a tumor bed is not necessarily an independent risk factor for recurrence.

It is important to note that the current body of literature emphasizes the high potential of ASCs in wound healing and regeneration. Multiple studies showed enhanced vascularization and improved wound healing after the treatment of skin wounds with ASC applications [62,63]. Highly promising findings for ASC application are also described in the context of spinal cord regeneration and recovery after partial hepatectomy [64,65]. Mostly, the paracrine effects of soluble factors that are secreted from ASCs are thought to be accountable for these effects, but direct cell interactions might also play an important role. Recently, it has been shown that ASCs, cultured in supernatant rich in growth factors, had enhanced specific binding capacity on selected cancer cells of fibrosarcoma and glioblastoma [66]. Menezes et al. observed that the presence of ASCs and not only their conditioned medium is necessary to build new maturated vessels [65]. Most likely, not only pro-angiogenetic factors such as VEGF, but also other growth factors are necessary to achieve tissue regeneration. ASCs might also execute immuno-suppressive effects in direct cell–cell interactions. Our group found that many ASCs express CD273/PD-L2. which plays a role in negative regulation of the adaptive immune response [13,67]. In HNSCC patients, in particular, functional and aesthetic reconstruction is challenging. Augmentation and regenerative therapy with cells that are capable of mediating rapid vascularization of grafts is highly appreciated. According to the present literature, mesenchymal stem cells promote regeneration, in addition to tumor cell growth of high-grade active tumors. Whether dormant tumor cells can be activated by ASCs in vivo remains unclear [61].

We performed the experiments of this study with two immortalized established cancer cell lines. The data show aspects of the interaction of active, proliferating tumor cells and ASC supernatants in vitro. Therefore, there is a limitation with respect to the comparability to the in vivo setting of lipotransfer after tumor resection. In this context, it is hard to prove whether dormant tumor cells can be activated by ASCs or their secretome. Other studies that used direct co-culture experiments of ASCs and HNSCC cells did not show increased migration or invasiveness in in vitro scratch assays and spheroid assays [35,38]. In this context, labeling of cells might influence cell migration and also only partially mimic the complex clinical setting with more cell types, e.g., immune cells involved. Another important limitation of this study and previous studies performed on the interaction of MSCs or ASCs and tumor cells is the lack of patient-specific tumor cell lines. Establishing both primary tumor cell and ASC cell lines from individual patients receiving tumor surgery and lipotransfer would help to overcome some of the limitations of the current studies.

Moreover, observational studies regarding the oncological safety of ASCs of lipotransfer procedures to the head and neck in head and neck cancer patients are necessary, e.g., in the framework of an augmentation of the vocal cord in laryngeal cancer. In such studies, cancer-specific outcome measures, such as the risk of relapse or disease-free survival, are necessary to evaluate the specific risk of stem cell applications in head and neck cancer patients. To date, most studies of autologous lipotransfer to the head and neck have focused on technical safety, efficacy in augmentation, and patient and surgeon satisfaction, and have relatively short periods of observational follow-up [68]. When adipose tissue transplantation is planned in cancer patients, the existence of residual or dormant squamous cell carcinoma cells should be at least excluded with intraoperative frozen section of surgical margins or, if it remains unclear, observation for locoregional recurrence after several months, e.g., 6 or 12 months, or an intraoperative frozen section.

## 5. Conclusions

Our data unambiguously show that ASC-conditioned media of different patients promote both tumor cell proliferation and migration of established cancer cell lines of the UAT, in addition to neo-angiogenesis of HUVECs in vitro. Therefore, ASCs are likely able to foster tumor growth and disease progression in squamous cell carcinoma via paracrine mechanisms. Although the literature indicates the topic remains controversial, this study highlights the potential tumor-promoting role of adipose tissue transplantation in head and neck cancer patients. However, further clinical trials are needed to evaluate the interaction of adipose-derived and other mesenchymal cells, and residual or dormant squamous cell carcinoma cells.

## Data Availability

The data presented in this study are available on request from the corresponding authors. The data are not publicly available due to restrictions of the institutional IRB statement in concordance to European/German legislation on data restriction.

## Supplementary Materials

The following are available online at https://www.mdpi.com/article/10.3390/cancers13112751/s1; Table S1: Patient characteristics.
