# Supplementary material for: Human Adipose-Derived Stem/Stromal Cells Promote Proliferation and Migration in Head and Neck Cancer Cells
Source: Cancers (Basel). 2021 Jun 1;13(11):2751. doi: 10.3390/cancers13112751 (PMC8199568; doi:10.3390/cancers13112751)
Supplement: Supplementary file 1 [file cancers-13-02751-s001.zip › cancers-1226872-supplementary.pdf]

Table S1: General patient characteristics

**Characteristics**		**Cohort (*n* = 5)**
**Gender**	Male	60%
	Female	40%
**Age at surgery [years]**		61.0±4.9
**Reason for local augmentation in the head and neck area**	Dysphonia	80%
	Cervical scarring	20%
**Body Mass Index [kg/m²]**		26.6±2.1
**Cancer in patient history**	Positive	60%
**Coagulapathy (e.g. medication-related)**	Positive	40%
